# Synthesis and Characterization of Gamma Radiation Induced Diallyldimethylammonium Chloride-Acrylic Acid-(3-Acrylamidopropyl) Trimethylammonium Chloride Superabsorbent Hydrogel

**DOI:** 10.3390/gels9020159

**Published:** 2023-02-16

**Authors:** Md Murshed Bhuyan, Jae-Ho Jeong

**Affiliations:** 1 Research Institute of Environment for Sustainability, Faculty of Engineering, Kyushu University, 744 Motooka, Nishi-Ku, Fukuoka 819-0395, Japan; 2 Thermal-Fluid Energy Machine Lab., Department of Mechanical Engineering, Gachon University, 1342, Seongnam-daero, Sujeong-gu, Seongnam-si 13120, Gyeonggi-do, Republic of Korea

**Keywords:** hydrogel, gamma radiation, superabsorbent, Diallyldimethylammonium Chloride

## Abstract

The gamma radiation technique is simple and time-saving for the synthesis of pure hydrogels. The present work focuses on synthesizing and characterizing Diallyldimethylammonium Chloride-Acrylic acid-(3-Acrylamidopropyl) trimethylammonium Chloride (DADMAC-AAc-APTAC) superabsorbent hydrogels. The hydrogels were synthesized by applying gamma radiation of different doses (2 kGy to 30 kGy) to two different compositions of monomers. The equilibrium swelling was found to be 33483.48% of dried gel for a 1:0.5:1 composition ratio of monomers at a 2 kGy radiation dose. Therefore, on the basis of equilibrium swelling, 2 kGy is the optimum radiation dose for synthesizing the hydrogel. Fourier transform infrared (FTIR), nuclear magnetic resonance (NMR) spectroscopy, and X-ray diffraction (XRD) characterization techniques were used to analyze and confirm the structure of the hydrogel. Thermogravimetric analysis (TGA) and Scanning electron microscopy (SEM) equipped with energy dispersive spectroscopy (EDS) clearly showed the thermal stability and surface morphology of the gel. Therefore, it can be concluded that hydrogels can be used in metal adsorption, drug delivery, and other fields of study.

## 1. Introduction

Hydrogels are three-dimensional polymeric networks prepared by physical or chemical cross-linking/grafting among monomers, or monomers and polymers that can retain a large amount of water in them without dissolving [1]. Ordinary hydrogels have many drawbacks such as lower equilibrium swelling and less efficiency in application sites [2]. If the hydrogels can swell and hold water more than 100 times their original weight, then it is referred to as a superabsorbent hydrogel (SH) [3,4]. There are various functional groups such as -NH_2_, -OH, -COOH, -CONH_2_, -CONH-, and -SO_3_H that are responsible for the swelling and hydrophilicity of the hydrogels [5]. The greater the number of those functional groups, the higher the equilibrium swelling leading to the super-absorbency [6,7]. SHs are extensively used in selective metal adsorption [8], drug delivery [9], agriculture [10], cell encapsulation [11], biosensors [12], etc. SHs are recently getting concerned with sanitary and hygiene applications studied by Peenal et al. [13]. Syed Sikandar Shah et al. reported that the SHs can also be incorporated with activated charcoal for selective adsorption of methylene blue dye which shows an excellent result [14]. In our previous work, we prepared APTAC-AAc hydrogel whose maximum equilibrium swelling was found to be 246 g/g of gel [15]. To improve the water absorbency and metal adsorption efficiency, the new monomer can be incorporated with the existing monomers. (3-Acrylamidopropyl) trimethylammonium chloride (APTAC) is a vinylic monomer with an ammonium chloride ionic part that can form superabsorbent hydrogel and facilitate the metal adsorption selectively from the multielement solution [16]. Acrylic acid (AAc) is the precursor of many polymeric hydrogels as well as a linking agent between two giant monomers or polymers [17]. Diallyldimethylammonium Chloride (DADMAC) is also a vinylic giant monomer containing tertiary ammonium chloride salt part which can be used for preparing ion exchangeable superabsorbent hydrogel copolymer [18]. Recently, its polymers were used as cationic adsorbent to adsorb negatively charged colloid materials [19], DNA carriers [20], in the paper and pulp industries [21], and industrial dye adsorption [22]. Due to water solubility, non-toxicity, hydrophilicity, and eco-friendly properties, it is an excellent candidate to be grafted or crossed-linked with other monomers resulting in the functional superabsorbent hydrogel. Tim B. Mrohs et al. prepared a superabsorbent hydrogel of DADMAC by using N, N-methylene bis acrylamide (BIS) cross-linker showing the maximum swelling capacity of 360 g/g gel and there are no studies on thermal stability [23]. Ziqing Tang et al. reported anionic dye adsorption by using DADMAC-based hydrogels which insists that further studies in increased swelling and application on metal adsorption [22]. Improvement of swelling of functional hydrogel is required to extend the efficiency of metal adsorption and release kinetics in drug delivery. Previously reported superabsorbent hydrogels show higher equilibrium swelling, but most of them are not functional (do not show selective adsorption to a specific metal). Congwei Li et al. prepared fluorescent chitosan hydrogel for selective detection and adsorption of Hg^2+^/Hg^+^. However, the hydrogels are not superabsorbent and the swelling ratio is not higher (~1.6) which limits the adsorption capacity [24]. Incorporation of DADMAC monomer with APTAC in hydrogel may increase the equilibrium swelling and adsorption efficiency. There are several ways of synthesis of hydrogel including chemical and radiation polymerization [25]. The Chemical method needs an initiator and cross-linking agent to proceed with the reaction where pure hydrogels cannot be obtained. On the other hand, radiation polymerization does not require an initiator or cross-linking agent for the polymerization among monomers resulting in pure hydrogels [26]. Gamma radiation is high energy (>5 keV, <0.25 A° wavelength, >12 EHz) electromagnetic radiation that can affect the properties of materials by producing free radicals leading to the formation of co-polymer [27]. One of the most used sources of gamma-rays are Co-60, which are not naturally abundant but can be produced by bombarding a Co-59 with a slow neutron [28]. The molecules containing double bonds interact with radiation in an aqueous medium to produce graft/cross-linked co-polymer through a free radical mechanism where no cross-linking agent and reaction initiator is needed [29]. All of the above-mentioned-monomers have a vinyl group in their structure which may easily interact to produce free radicals resulting in the graft/cross-linked co-polymer. Recently, Ion Calina et al. synthesized superabsorbent hydrogel from xanthan gum/Sodium carboxymethylcellulose/graphene oxide by applying e-beam radiation where the highest swelling degree is 6000% only at a higher radiation dose of 15 kGy [7]. Abdul Haleem et al. also reported gamma radiation-induced hydrophobic cryogels for the adsorption of organic solvents and oils [30]. However, the superabsorbent hydrogel from the combination of DADMAC and APTAC has yet not been studied by applying gamma radiation. The main objective of the present work is to synthesize DADMAC-AAc-APTAC superabsorbent hydrogel by applying different doses of gamma radiation and then characterization.

## 2. Results and Discussion

### 2.1. Radiation Polymerization of DADMAC-AAc-APTAC

**Scheme 1:**
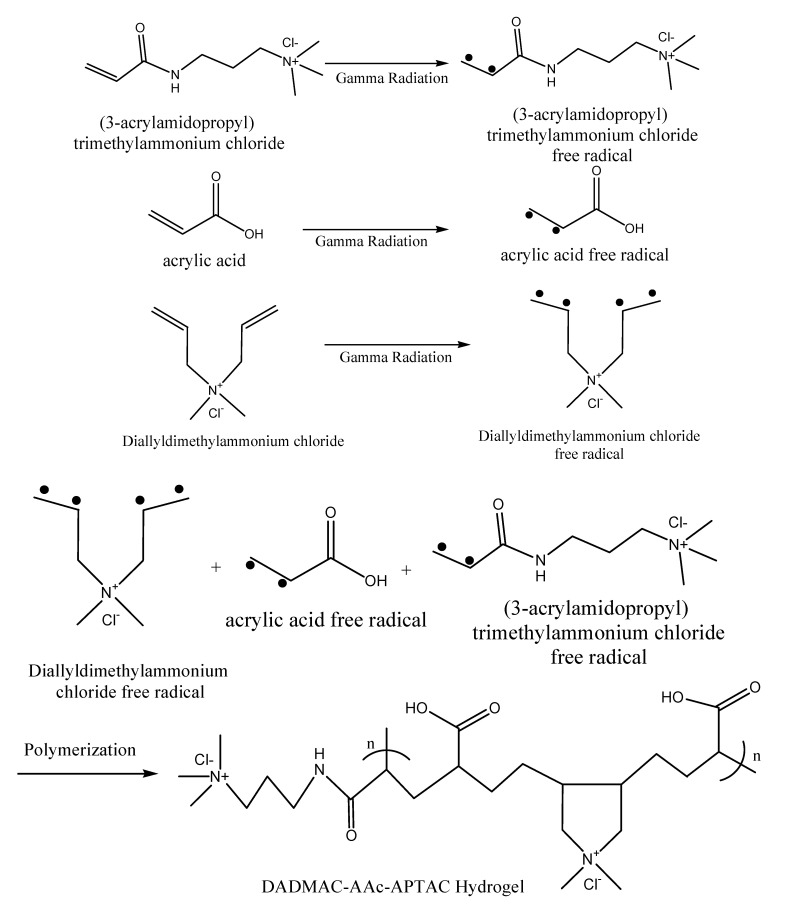
Probable polymerization of DADMAC-AAc-APTAC hydrogel.

Radiation polymerization gives a pure yield because of not using the cross-linking agent and initiator. In this work, radiation from the Cobalt-60 source was applied and synthesized gels. Gamma radiation interacts with the vinyl part of the raw materials to produce free radicals which propagate and terminate to yield the final polymeric gel. Gamma radiation was irradiated on the blend solution of different compositions of monomers. The solution of DADMAC and APTAC does not give directly hydrogel products upon irradiation. However, in presence of AAc, they undergo gel formation which can be attributed to the bulky groups of APTAC and DADMAC hindering the movement of monomers to get close proximity and collision for proceeding with the reaction [31]. Small group AAc is turned into free radicals and acts as a linker between two big crowded monomers [32]. Scheme 1 shows the probable radiation polymerization in aqueous media.

### 2.2. Effect of Radiation Dose on APTAC-AAc-DADMAC Gel Content

**Figure 1:**
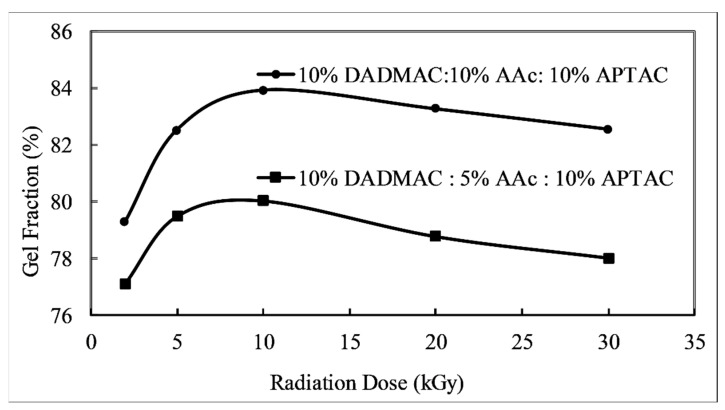
Effect of radiation dose on gel fraction of hydrogel.

Gel fraction is the amount of gel produced and extracted by removing unreacted contaminants from the gel network. Figure 1 shows the effect of radiation dose on the gel production during the reaction among monomers. The figure indicates the smaller gel production at a lower dose following the increasing trend till 10 kGy, then starts decreasing [33]. At lower radiation doses, all the particles of raw materials cannot be activated for the reaction which lessens the gel fraction [34,35]. The figure also reflects the effect of the concentration of monomers where the higher concentration (10%) of acrylic acid in the blend solution gives a higher gel fraction compared to the lower concentration (5% acrylic acid). This is due to the greater number of grafting and cross-linking among the monomers as a larger number of molecules feel irradiation. Owing to the steric hindrance, all of the free radicals of larger monomers cannot propagate to produce their own homo-polymer. Acrylic acid is a smaller monomer than APTAC and DADMAC, which is why it can link the big monomers giving the cross-linked and grafted copolymers.

### 2.3. Effect of Radiation Dose on Equilibrium Swelling of Gels

**Figure 2:**
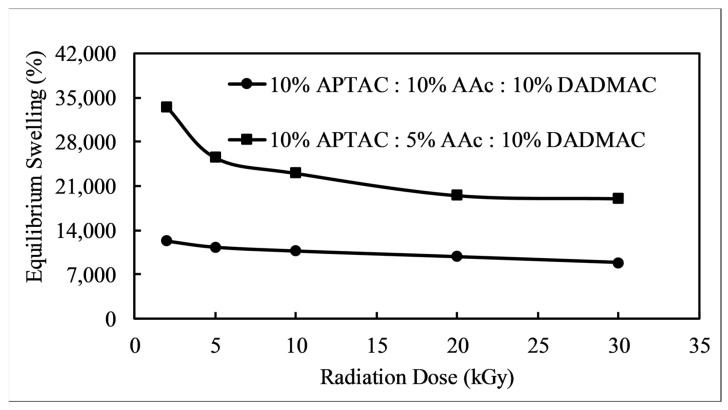
The effect of gamma radiation dose on equilibrium swelling of the hydrogel at room temperature.

**Figure 3:**
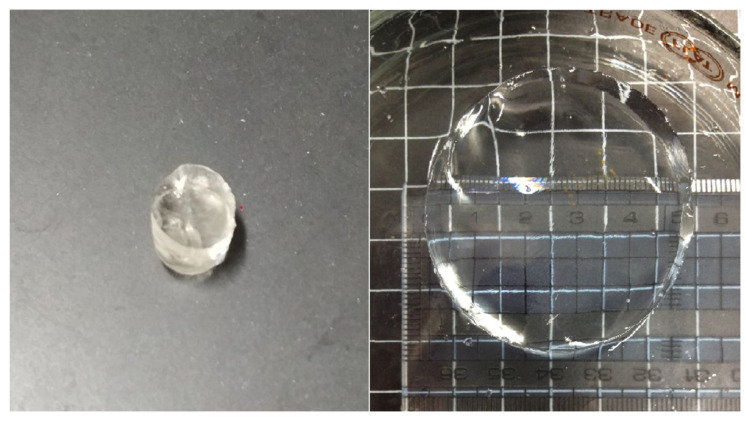
DADMAC-Aac-APTAC hydrogel before and after swelling.

The equilibrium swelling of hydrogels is the most important and significant property of hydrogel as many applications depend on it. Figure 2 represents the equilibrium swelling of hydrogels of both compositions as the function of the radiation dose applied for synthesizing gels. Both the composition ratio 1:1:1 and 1:0.5:1 of DADMAC: AAc: APTAC show a swelling percentage of about 12,300.23% and 33,483.48% for the radiation dose of 2 kGy which follows the declining trends up to 30 kGy radiation dose. At higher radiation doses, a greater number of monomers become activated to make the network of polymer denser. Moreover, the concentration of acrylic acid affects elaborately on the water absorption can be attributed to the lower amount of acrylic acid linker making wider void space inside the hydrogel networks. In the composition of a 1:0.5:1 ratio, a lower concentration of acrylic acid facilitates higher swelling. Thus, the swelling is higher for lower radiation dose (2 kGy) and acrylic acid content (5%). The author previously reported on APTAC-AAc superabsorbent hydrogel having ~24,600% equilibrium swelling at neutral pH (6.5~7.5). In this study, the hydrogels are showing better swelling. Figure 3 exhibits the hydrogel before and after swelling in water at room temperature where the swelling behavior is observed.

### 2.4. Characterization of Hydrogel by FTIR Spectroscopy

**Figure 4:**
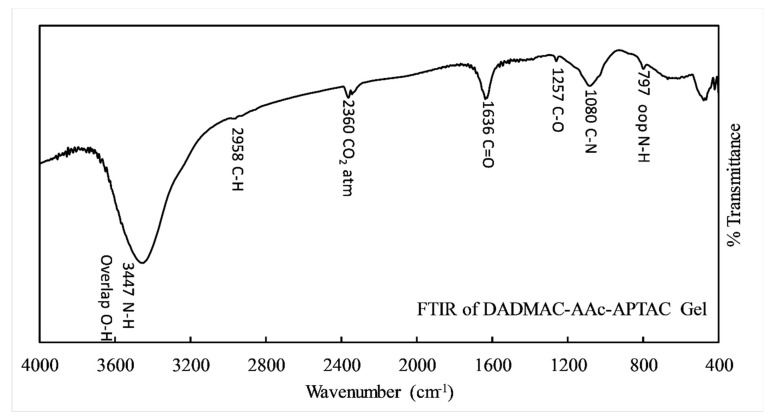
FTIR spectrum of DADMAC-AAc-APTAC hydrogel.

FTIR spectroscopy of DADMAC-Aac-APTAC gel was measured by using the KBr reference. Figure 4 Presents the spectrum of gel which indicates the peak at 3447 cm^−^^1^ for N-H stretching of secondary amide overlapping with O-H of carboxylic acid. The peak at 2958 cm^−^^1^ is for C-H stretching. The other characteristic peaks at 1636 cm^−^^1^ for –C=O of tertiary amide, 1257 cm^−^^1^ (medium intensity) for C-O of carboxylic acid, 1080 cm^−^^1^ for –C-N stretching of tertiary amide and 797 cm^−^^1^ for -N-H out of plane bending [36]. Thus, the presence of –N-H and –C-N peaks belong to the amide group which indicates the copolymerization between DADMAC and APTAC monomers via acrylic acid linkage.

### 2.5. X-ray Diffraction Analysis

**Figure 5:**
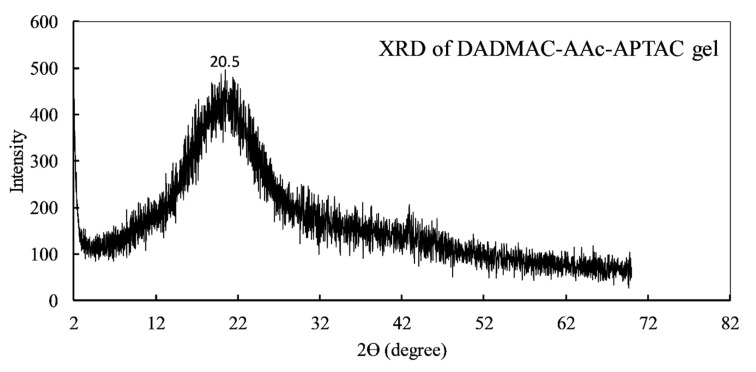
XRD spectrum of DADMAC-AAc-APTAC hydrogel.

Whether the hydrogel is crystalline or amorphous was examined with an X-ray diffraction pattern run with a scene rate of 2 °C over a 2θ range of 2 °C to 70 °C. Figure 5 shows the X-ray diffraction graph where the broad peak at 20.5 °C indicates the amorphous structures of the hydrogel. The presence of the quaternary ammonium group hinders the formation of the crystal structure of the polymer resulting in the amorphous structure of the DADMAC-AAc-APTAC gel. Furthermore, the hydrogen bonding broad peak 2θ = 12 °C to 32 °C corresponds to the coherent diffraction of the cross-linking network [37]. Since there is no obvious diffraction peak in the range of 2θ = 2 °C to 70 °C, so, the hydrogel belongs to the amorphous structure.

### 2.6. Nuclear Magnetic Resonance (NMR) Spectroscopy

**Figure 6:**
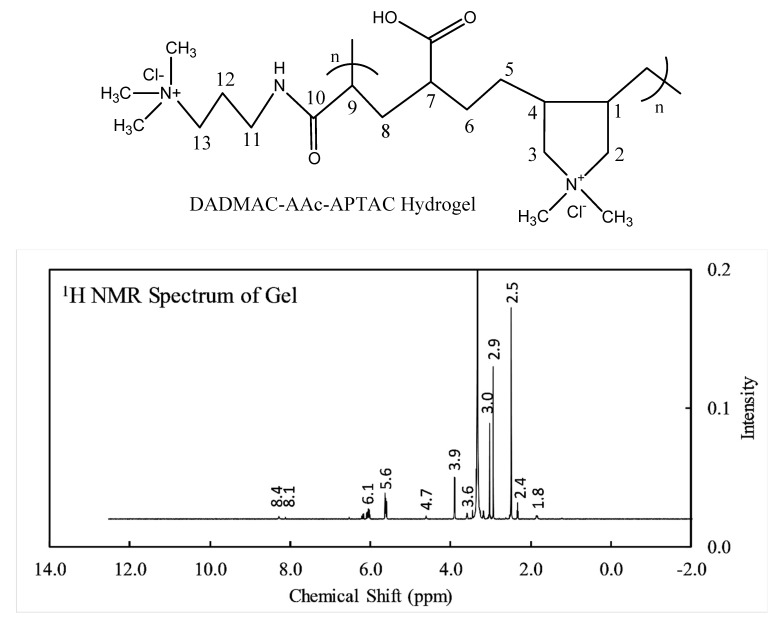
^1^H NMR of DADMAC-AAc-APTAC hydrogel.

Proton nuclear magnetic resonance (^1^H NMR) spectrum hydrogel was measured in dimethyl sulfoxide (DMSO) solvent for studying the different environments of the proton of the gel. ^1^H NMR spectrum supports the other analysis methods in confirming the preparation of hydrogel. Hydrogel displays the following corresponding signals (Figure 6): The peak at 1.8 ppm corresponds to methylene (-CH_2_) proton (carbon no. 2,3,6,8), at 2.4 ppm for –CH- proton of ring part of DADMAC (carbon no. 1, 4), at 2.5 ppm for –CH_2_- of propyl group of amide (carbon no. 12), doublet peak at 2.9 ppm and 3.0 ppm are for the proton of quaternary ammonium part of DADMAC (-CH_3_)_2_ and APTAC (-CH_3_)_3_, respectively, peak at 3.6 ppm and 3.9 ppm for –NH-CH_2_ (carbon no. 11) and ^+^N-CH_2_ (carbon no. 13) protons. The doublet peaks at 5.6 ppm and 6.1 ppm are for –CH of the APTAC-AAc chain (carbon no. 7 and 9). The proton of –NH and –COOH show peaks at 8.1 ppm and 8.4 ppm [36,38,39,40]. Thus, the ^1^H NMR supports the FTIR in confirming the preparation of DADMAC-AAc-APTAC hydrogel.

### 2.7. Thermogravimetric Analysis (TGA)

**Figure 7:**
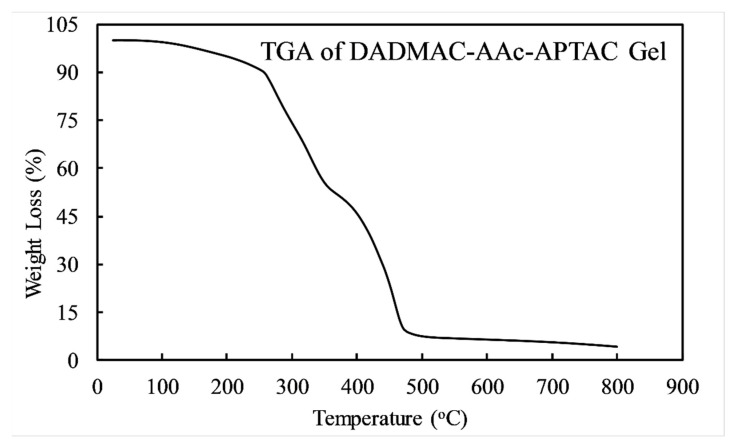
TGA graph of DADMAC-AAc-APTAC gel prepared by 2 kGy radiation dose.

Thermal analysis is important to know the thermal stability over a range of temperatures indicating whether the gels are applicable in different fields of study. To know the thermal change in the hydrogel, thermogravimetric analysis was run at a 10 °C scene rate over a temperature range from 25 °C to 800 °C. Figure 7 illustrates the weight loss (%) of hydrogel through two stages as a function of temperature increases gradually. In the first stage, temperature changes from 25 °C to 150 °C due to the releasing of moisture from the void space of hydrogel with a weight loss of 3% of the original weight. The second stage represents the polymer degradation from 150 °C to 480 °C temperature for the degradation of carbonaceous products where the mass loss is 92% and the residual mass of 8%. The maximum degradation was observed at 471 °C [37]. Therefore, the hydrogel is thermally stable enough to use in different fields of study.

### 2.8. Surface Analysis by SEM-EDS

**Figure 8:**
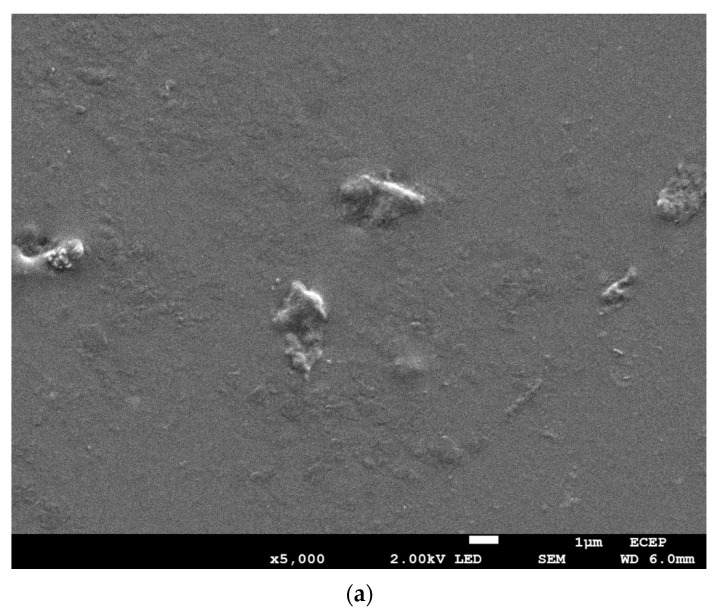

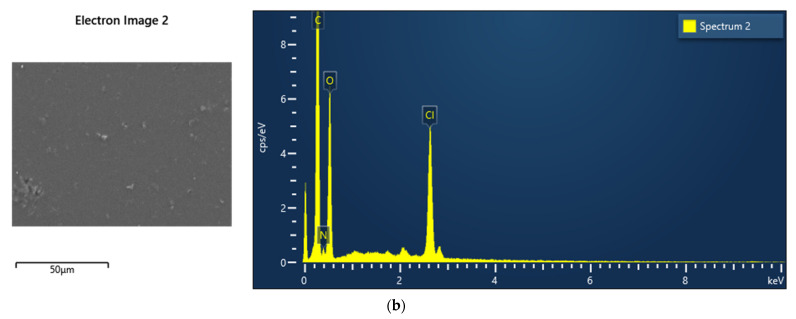
(**a**) SEM and (**b**) EDS of DADMAC-AAc-APTAC hydrogel.

SEM-EDS images of DADMAC-AAc-APTAC hydrogel prepared by 2 kGy radiation dose and 1:0.5:1 composition are illustrated in Figure 8. Figure 8a shows SEM of gel with a smooth surface morphology bearing an entangled network of hydrogel polymer. Figure 8b represents the significant constituent elements of the hydrogel are Carbon (C), Nitrogen (N), Oxygen (O), and Chlorine (Cl) of quaternary ammonium salt, and the compositional percentage is listed in Table 1. Therefore, it can be concluded that the monomers have undergone polymerization perfectly without disrupting their structure.

## 3. Conclusions

In this work, DADMAC-AAc-APTAC superabsorbent hydrogels were prepared successfully by applying various doses (2 kGy to 30 kGy) of gamma radiation. The gel fraction was found maximum at 10 kGy radiation dose for both compositions of monomers. Since the aim of this work is to prepare superabsorbent hydrogel, the radiation dose was optimized on the basis of equilibrium swelling which was found to be 2 kGy for both compositions. The composition 10% DADMAC: 5% AAc: 10% APTAC gives the best equilibrium swelling result at pH 6.5~7.5 is about 33,483.48% of the dried weight of the gel at room temperature. The copolymerization between DADMAC and APTAC was confirmed by FTIR and NMR spectroscopic analysis. XRD showed the amorphous structure of the hydrogel and thermogravimetric analysis (TGA) revealed thermal stability. SEM-EDS showed a smooth surface and significant elements of the gel structure. So, the hydrogels can be prepared at lower radiation doses (2 kGy) for application in different fields of study such as metal adsorption, drug delivery, etc.

## 4. Materials and Methods

### 4.1. Materials and Reagents

(3-Acrylamidopropyl) trimethylammonium chloride, Diallyldimethylammonium Chloride, and Acrylic Acid were purchased from Sigma Aldrich, Germany. All samples were prepared using ultra-pure water and the temperature was kept at 198 K for the experiments. The pH of the solutions was maintained by using nitric acid (HNO_3_) and ammonium hydroxide (NH_4_OH).

### 4.2. Apparatus and Instruments

Functional groups of DADMAC-AAc-APTAC hydrogel were analyzed by FTIR spectroscopy (Thermo Scientific Nicolet iS50R FT-IR). Proton and Carbon NMR were performed by using JMTC-500/54/JJ. Measurement of thermal properties of a pre-dried gel was carried out by Thermogravimetric analysis(TGA) (TGA 8000, PerkinElmer, Waltham, USA) under a continuous N_2_ gas flow and heating rate of 10 °C/min. Whether the hydrogel is crystalline or amorphous was confirmed by X-Ray Diffraction (XRD, Rigaku Smart Lab, Tokyo, Japan (Lamda = 1.54059 Angstrom)) analysis. The surface morphology of dried hydrogels was observed with an SEM (JEOL, JSM-7900F) equipped with an EDS and with platinum coating.

### 4.3. Synthesis of DADMAC-AAc-APTAC Hydrogels by Gamma Radiation

An aqueous blend solution of DADMAC, AAc, and APTAC was prepared by mixing their individual solution in a round bottom flask with stirring at 500 rpm and room temperature. The different ratio of the monomers was maintained to optimize the better products shown in Table 2. The mixed solution was then taken in glass tubes followed by the passing of N_2_ gas to remove air from the tube. The samples were subjected to irradiation with a gamma source (Co-60) at different radiation doses (kGy) ranging from 2 to 30 kGy (Table 3) over a certain period of time. The Co-60 gamma source is the point source that emits different radiation doses as a function of irradiation time and distance between the sample and the gamma source. After irradiation, the hydrogels were collected, cut into small pieces, and dried at 50 °C temperature.

### 4.4. Post-Synthesis Analysis

#### 4.4.1. Extraction and Measurement of Gel Content

The dried and weighed gels were extracted in water at 40 °C by keeping the gels in a beaker containing ultra-pure water for 24 h. After 24 h of soaking, the samples were taken and dried in an oven at 40 °C to constant weight. During extraction, the unreacted monomers and unwanted contaminants leave the hydrogel network. From the two weights of dried gels, (before and after extraction) the gel content was calculated by using the following equation:

(1) $$\mathrm{Gel}\;\mathrm{fraction}\;\left[\%\right]=\frac{W_{1}}{W_{0}}\times 100\%$$

where *W*_0_ and *W*_1_ are the dried-gel weights before extraction and after extraction, respectively.

#### 4.4.2. Measurement of Super-Absorbency at Equilibrium Swelling

Equilibrium swelling was measured by keeping the dried hydrogels in aqueous media of neutral pH (6.5~7.5) at room temperature. After 24 h, the samples were taken out and blotted with filter paper and weight. To check the constant weight, the samples were kept soaking for up to 26 h and found no change in weight. The equilibrium swelling was evaluated from the dried and swelled weight of hydrogels by using the following equation:

(2) $$\mathrm{Water}\;\mathrm{absorption}\;\left[\%\right]=\frac{W_{t}-W_{1}}{W_{1}}\times 100\%$$

where *W*_1_ and *W_t_* are the dried-gel weight and the gel weight after swelling in the solution respectively. The water absorption was repeated three times.

## Figures and Tables

**Table 1:** Composition of significant elements of hydrogel.

Element	Weight %	Weight % Sigma
Carbon (C)	59.15	0.59
Nitrogen (N)	7.55	0.76
Oxygen (O)	28.38	0.40
Chlorine (Cl)	4.92	0.08
Total:	100	

**Table 2:** Different composition of raw materials and observation after gamma irradiation.

Composition of Raw Materials	Observation
DADMAC (10%) + APTAC (10%)	No hydrogel found
DADMAC (10%) + AAc (10%) + APTAC (10%)	Solid Hydrogel found
DADMAC (10%) + AAc (5%) + APTAC (10%)	Solid Hydrogel found

**Table 3:** Radiation dose and respective gel products for APTAC + AAc + DADMAC.

Radiation Dose (kGy)	2	5	10	20	30
Gel Product	Solid gel	Solid gel	Solid gel	Solid gel	Solid gel

## Data Availability

BHUYAN: MD (2022), “DADMAC-AAc-APTAC superabsorbent Hydrogel”, Mendeley Data, V1, https://doi/org/10.17632/b47zht4×2t.1 (accessed on 5 February 2023).
